# Deep Learning for Predicting Distant Metastasis in Patients with Nasopharyngeal Carcinoma Based on Pre-Radiotherapy Magnetic Resonance Imaging

**DOI:** 10.2174/1386207325666220919091210

**Published:** 2023-03-24

**Authors:** Hong-Li Hua, Yu-Qin Deng, Song Li, Si-Te Li, Fen Li, Bai-Kui Xiao, Jin Huang, Ze-Zhang Tao

**Affiliations:** 1 Department of Otolaryngology-Head and Neck Surgery, Renmin Hospital of Wuhan University, 238 Jie-Fang Road, Wuhan, Hubei 430060, P.R. China;; 2 College of Mathematics and Computer Science, Wuhan Textile University, No.1 Fangzhi Road, Wuhan, Hubei, 430200, P.R. China;; 3 Research Institute of Otolaryngology-Head and Neck Surgery, Renmin Hospital of Wuhan University, 238 Jie-Fang Road, Wuhan, Hubei 430060, P.R. China;; 4 Department of Otolaryngology-Head and Neck Surgery, Central Laboratory, Renmin Hospital of Wuhan University, 238 Jie-Fang Road, Wuhan, Hubei 430060, P.R. China

**Keywords:** Deep learning, transfer learning, nasopharyngeal carcinoma, distant metastasis, peritumour, cervical metastatic lymph node (CMLN)

## Abstract

**Importance:**

Accurate pre-treatment prediction of distant metastasis in patients with Nasopharyngeal Carcinoma (NPC) enables the implementation of appropriate treatment strategies for high-risk individuals.

**Purpose:**

To develop and assess a Convolutional Neural Network (CNN) model using pre-therapy Magnetic Resonance (MR) imaging to predict distant metastasis in NPC patients.

**Methods:**

We retrospectively reviewed data of 441 pathologically diagnosed NPC patients who underwent complete radiotherapy and chemotherapy at Renmin Hospital of Wuhan University (Hubei, China) between February 2012 and March 2018. Using Adobe Photoshop, an experienced radiologist segmented MR images with rectangular regions of interest. To develop an accurate model according to the primary tumour, Cervical Metastatic Lymph Node (CMLN), the largest area of invasion of the primary tumour, and image segmentation methods, we constructed intratumoural and intra-peritumoural datasets that were used for training and test of the transfer learning models. Each model’s precision was assessed according to its receiver operating characteristic curve and accuracy. Generated high-risk-related Grad-Cams demonstrated how the model captured the image features and further verified its reliability.

**Results:**

Among the four models, all intra-peritumoural datasets performed better than the corresponding intratumoural datasets, with the CMLN intra-peritumoural dataset exhibiting the best performance (average area under the curves (AUCs) = 0.88). There was no significant difference between average AUCs of the Max and NPC tumour datasets. AUCs of the eight datasets for the four models were higher than those of the Tumour-Node-Metastasis staging system (AUC=0.67). In most datasets, the xception model had higher AUCs than other models. The efficientnet-b0 and xception models efficiently extracted high-risk features.

**Conclusion:**

The CNN model predicted distant metastasis in NPC patients with high accuracy. Compared to the primary tumour, the CMLN better predicted distant metastasis. In addition to intratumoural data, peritumoural information can facilitate the prediction of distant metastasis. With a larger sample size, datasets of the largest areas of tumour invasion may achieve meaningful accuracy. Among the models, xception had the best overall performance.

## 1 INTRODUCTION

Nasopharyngeal Carcinoma (NPC) is one of the most prevalent malignancies in southern China and Southeast Asia [1, 2]. However, its anatomical location makes it inaccessible, and radiotherapy is the primary treatment for NPC because it is extremely sensitive to radiation. In recent years, intensity-modulated radiotherapy has replaced conventional traditional radiotherapy. Intensity-modulated radiotherapy improves local recurrence-free survival [3], especially in patients with advanced NPC. According to prior studies, the locoregional control rate is more than 90%, and the major cause of treatment failure is the occurrence of Distant Metastasis (DM) [4, 5]. Most NPC patients develop cervical lymph node metastasis and DM. The vast majority of DM occurs 3 years after completion of radiotherapy; approximately half of the patients present with metastasis within 1 year, while a quarter present in the second and third years [6]. Although improvements in radiotherapy and chemotherapy have increased the survival rate of patients with DM, the 5-year survival rate remains < 10% [7]; furthermore, most patients die earlier as metastasis progress. Thus, identifying high-risk patients with DM before commencing more aggressive therapeutic strategies is necessary.

Currently, oncologists select treatment methods for NPC patients based on their Tumour-Node-Metastasis (TNM) stage [8]. However, patients with the same TNM stage can have different outcomes even when they receive the same treatment [9]. An explanation could be that the TNM stage reflects only the anatomical differences in the types of tumour invasion while neglecting intratumoural variations. Various studies predicted the risk of DM in NPC patients based on molecular mechanisms. Epstein-Barr (EB) virus DNA load, neutrophil-to-lymphocyte ratio, pre-treatment serum EB virus shell antigen IgA antibody levels, microRNA signatures, and epidermal growth factor receptor overexpression are factors related to metastasis and prognoses [10-14]. However, none of these indicators have high predictive specificity and accuracy.

With recent and rapid advancements in artificial intelligence technology, Deep Learning (DL) algorithms can efficiently analyse and process large datasets of medical images. These algorithms can play key roles in preoperative diagnoses, evaluation of curative effects, and determination of prognoses of NPC [15-18]. Recent studies demonstrated that the diagnostic accuracy of a DL-based NPC detection model is significantly higher than that of imaging experts; also, it can automatically outline the primary focus of the nasopharynx to achieve accurate tumour segmentation [13, 19-21]. Some studies compared the prognostic accuracy of a Magnetic Resonance Imaging (MRI)-based DL model to that of the TNM system, reporting that the accuracy of the DL model in predicting overall survival, DM-free survival, and locoregional recurrence-free survival in NPC patients was higher than that of the TNM system-based model [15, 16]. These findings indicate that the DL model can effectively predict the curative effect in NPC patients. However, the use of a DL model to predict DM of NPC is yet to be established. Some previous studies on DM prediction in NPC patients were based on radiomics approaches [6, 22, 23]. A study was based on DL; however, their dataset comprised less than 200 images; hence this model’s accuracy may be unideal [24]. Predicting the risk of DM and providing individualised and accurate treatment suggestions before metastasis can occur is key to improving the therapeutic effect in NPC patients and achieving long-term survival. This study aimed to establish a pre-treatment DL model using MR images of the nasopharynx and neck to predict the risk of DM, facilitating a more rigorous screening and intensive treatment strategy for high-risk patients, thus improving treatment efficacy and survival rates among NPC patients.

## 2 MATERIALS AND METHODS

### 2.1 Patient Screening

**Fig. (1):**
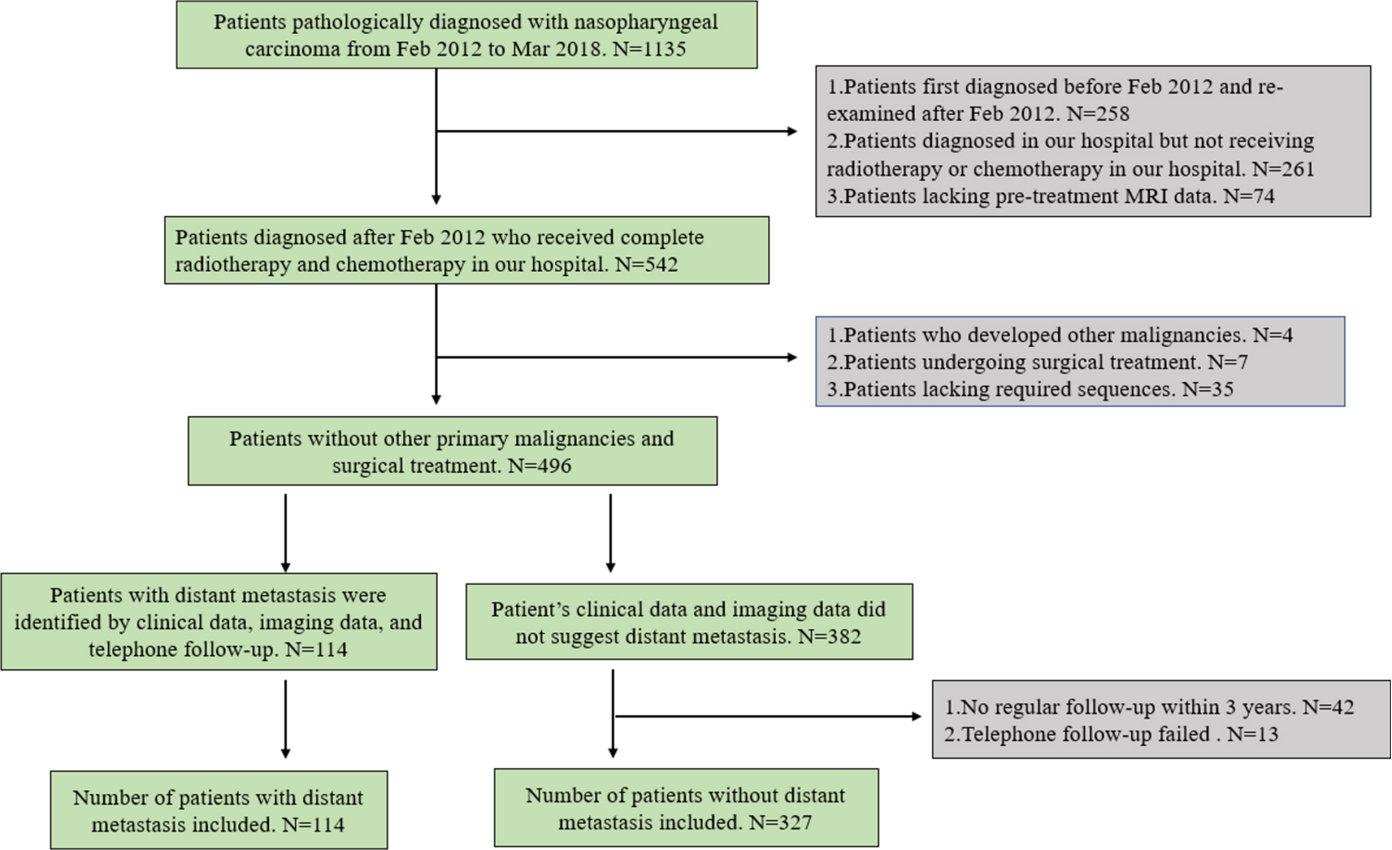
Flowchart of the patient selection process.

In total, 441 patients with primary NPC diagnosed and treated at the Renmin Hospital of Wuhan University between February 2012 and March 2018 were retrospectively selected. Data on patients’ age, sex, clinical TNM stage, treatment modality, pathological results, and pre-treatment MR images were collected together with other data. The TNM staging was based on the 7th American Joint Committee on Cancer manual [25]. Patients were routinely examined using a bone scan, chest Computed Tomography (CT), abdominal ultrasound, and MRI and were diagnosed with DM if one of the tests confirmed its presence. If the imaging diagnosis was unclear, the suspected site was biopsied, or a dynamic periodic review was performed every 3 months for at least 12 months. If the lesion further enlarged, the patient was considered to have DM. If no significant change in the lesion was observed during the follow-up period, DM was excluded. Since more than 90% of patients develop DM within 3 years of initial NPC diagnosis, we followed up with patients without DM for at least 3 years. When data was collected, we observed that some patients who did not return to our hospital were diagnosed with DM at local hospitals. Therefore, in addition to ensuring regular follow-ups for 3 years, we also ensured strict telephone follow-ups for all patients and excluded patients lost to the telephonic follow-up. Patients were only classified as not having DM when all clinical, imaging, and telephonic follow-up data indicated that the patient had no DM. When any of these data indicated the presence of DM, we classified patients into the DM group. The inclusion criteria were as follows: primary NPC pathologically diagnosed and treated at our hospital; MRI examination of the nasopharynx within 1 month before treatment; bone scan, chest CT, abdominal ultrasound, and MRI data; received regular reviews; and complete and uninterrupted treatment. The exclusion criteria were as follows: the presence of a primary malignant tumour at other sites, clinical uncertainty as to whether metastasis occurred, surgery included in the treatment plan, failed telephonic follow-ups, interrupted treatment owing to intolerance to radiotherapy or chemotherapy, or lack of required imaging data. Patient screening and the exclusion criteria are shown in the flowchart (Fig. **1**).

### 2.2 Image Acquisition and Pre-Processing

All patients underwent nasopharynx and neck imaging with a 1.5-T MR scanner providing pre-treatment axial T1-weighted enhanced images, which were stored in DICOM format at a size of 512 × 512 pixels. Segmenting the Region of Interest (ROI), axial T1-weighted enhanced images obtained from the nasopharynx and neck scans were imported into Adobe Photoshop software. The rectangular area closest to the tumour tissue was manually delineated, layer by layer, all segregated by an experienced radiologist. The approximate size of the ROI, including all tumour tissues and cervical metastatic lymph nodes, was 400 × 200 pixels in each layer of the image; however, the size of the rectangular ROI varied according to tumour size. We used the Padding operation in the convolutional neural network (CNN) to fill the “0” pixels around the image and convert all segmented images into 512 × 512 pixels. An NPC-CMLN dataset was established based on the segregated ROIs of primary tumours and Cervical Metastatic Lymph Nodes (CMLNs) in NPC patients to compare the role of the primary tumour and CMLNs in predicting DM. The NPC and CMLN datasets were established based on the primary tumour and CMLNs, respectively. Since tumour size may also provide important information for predicting DM, we selected three slices of the largest tumour invasion areas to establish a Max tumour dataset. As reported, the peritumoural region has diagnostic value for predicting the tumour state [26]; therefore, we also enlarged the ROI of the tumour by 3 mm to include the peritumoural area, defined as the intra-peritumoural area of the tumour. Each of the above four datasets included the intratumoural and intra-peritumoural sub-datasets, comprising eight datasets in total: NPC-CMLN intratumour, NPC intratumour, CMLN intratumour, Max tumour intratumour, NPC-CMLN intra-peritumour, NPC intra-peritumour, CMLN intra-peritumour, and Max tumour intra-peritumour. Patients were divided into the metastatic and nonmetastatic groups according to the presence or absence of metastasis during the follow-up period, and each MR image was labelled according to the patient’s results. In total, 441 NPC patients were assigned to the training and test groups in a 4:1 ratio based on a random number table.

### 2.3 Network Architecture and Model Development

**Fig. (2):**
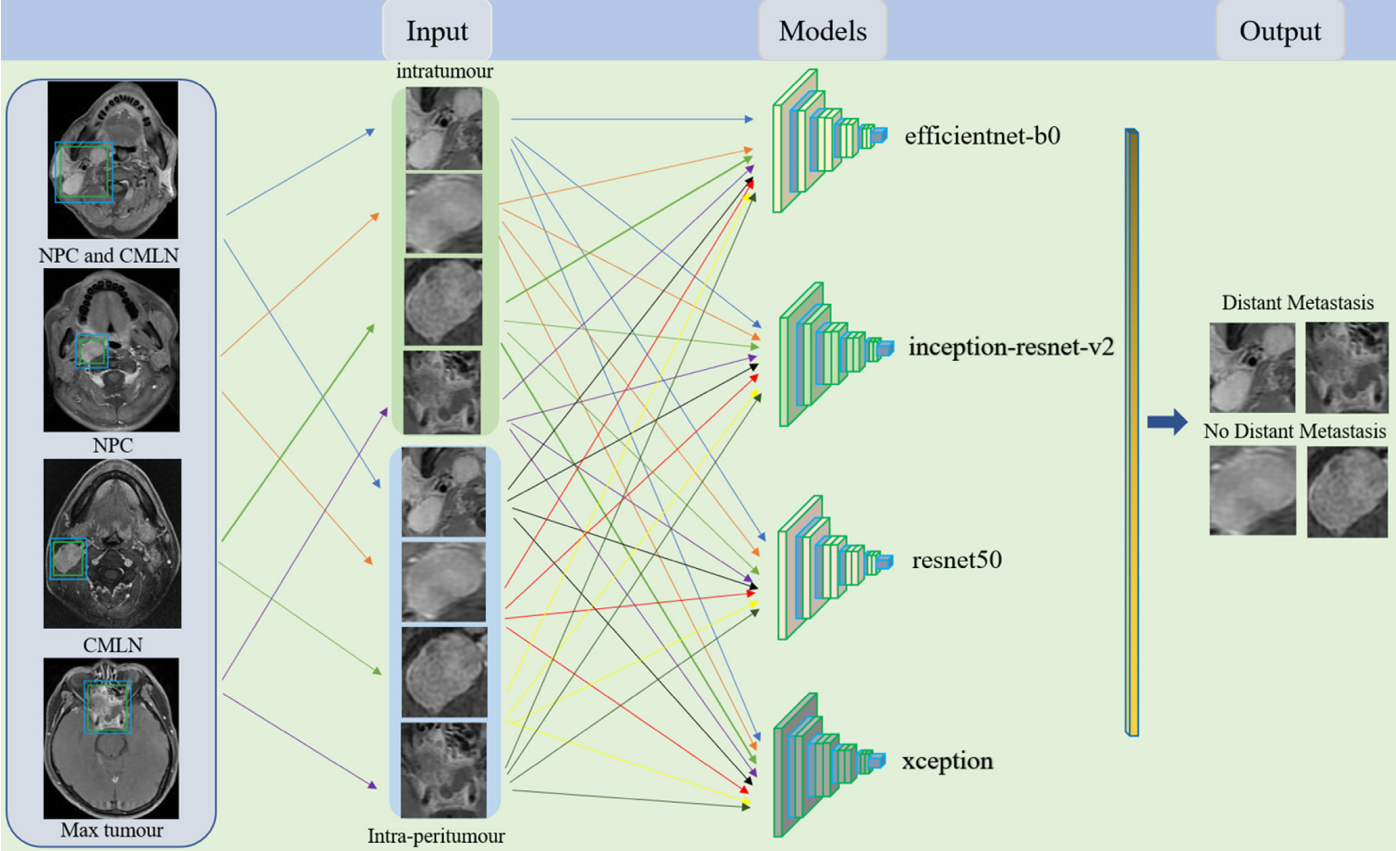
Models for the prediction of DM.

We developed an end-to-end DL framework based on the MR images of the nasopharynx and neck to predict DM using Keras 2.2.0 and TensorFlow 2.0 in Python version 3.6. The DL network comprises convolution, activation, pooling, batch normalisation, and zero padding layers, which complete feature extraction, nonlinear feature acquisition, representative feature selection, model training acceleration, and image size unification during model training, respectively. We transferred the efficient net-b0, inception-resnet-v2, resnet50 and xception CNN models frequently used in recent years for training. As the base network in the efficient net series, efficientnet-b0 comprises 16 MBconv blocks, two convolutional layers, a global average pooling layer and a fully connected layer, with the smallest model size and fastest detection speed. The inception-resnet-v2 network consists of a stem, Inception -resnet blocks and a prediction layer, characterized by the introduction of resnet residual structure in the inception module and the integration of the advantages of the two modules, thus improving the performance of model image recognition. Resnet50 is composed of 49 convolutional layers and one fully connected layer. In this residual network, a convolutional neural network with a depth of 50 layers is constructed by stacking 16 residual blocks, so the network has a stronger feature extraction capability for images. Xception is an improved network proposed by Google for inception3, which introduces deep separable convolution and residual join to reduce the number of model parameters while maintaining high accuracy and solving the problem of gradient disappearance or explosion. The eight datasets were used to train each model (Fig. **2**). Finally, 32 training models were obtained. We used transfer learning to overcome the limited number of training images. We modified the last layer of the CNN, mainly deleting its top layer and adding a customised full connection layer on the top layer. We used the Adam Optimizer for network training. The training aimed to optimise the DL model parameters and establish a connection between MR images and the DM. The model training process is an iterative process where the function of each iteration is to optimise the model until the best prediction performance is obtained. We used cross entropy as the cost function to evaluate the model’s predictive performance. The batch size and initial learning rate were set as 32 and 0.0001, respectively, and training was stopped after 40 epochs of model training. We set the dropout rate for the full link layer as 0.5 and added regular terms to the objectivefunction to prevent overfitting. To further clarify the principles and explain the prediction results of the DL model, we used a visualisation algorithm to demonstrate how the DL model learned the DM-related information. The training queue contained approximately 80% of the dataset (354/441 patients), while the test queue contained 20% (87/441).

### 2.4 Statistical Analysis

Statistical analyses for clinical data comparison were performed using SPSS (version 24.0) and Python (version 3.6). The Kolmogorov-Smirnov test was used to verify the normality of data distribution. Continuous variables conforming to a normal distribution were expressed as means ± standard deviations. The chi-square (χ^2^) test was used to compare the frequency data, while the t-test used continuous data. The areas under the Receiver Operating Characteristic (ROC) curves (AUCs), accuracy, sensitivity, specificity and F1-score were calculated to evaluate the performances of the eight datasets and the TNM staging system. Differences were considered statistically significant at *P* < 0.05. We demonstrated the prediction process of the model and its reliability by extracting feature maps from the final convolutional layer and generating Grad-Cams for high-risk areas of the image using the Matplotlib package in Python.

## 3 RESULTS

### 3.1 Clinical Characteristics of the Study Population

After screening, 441 patients who met the eligibility criteria were enrolled in the primary (N = 354) and test (N = 87) cohorts. There were no significant differences in the general information between the training and test groups Table **1**.

### 3.2 Performance of the Models

**Fig. (3):**
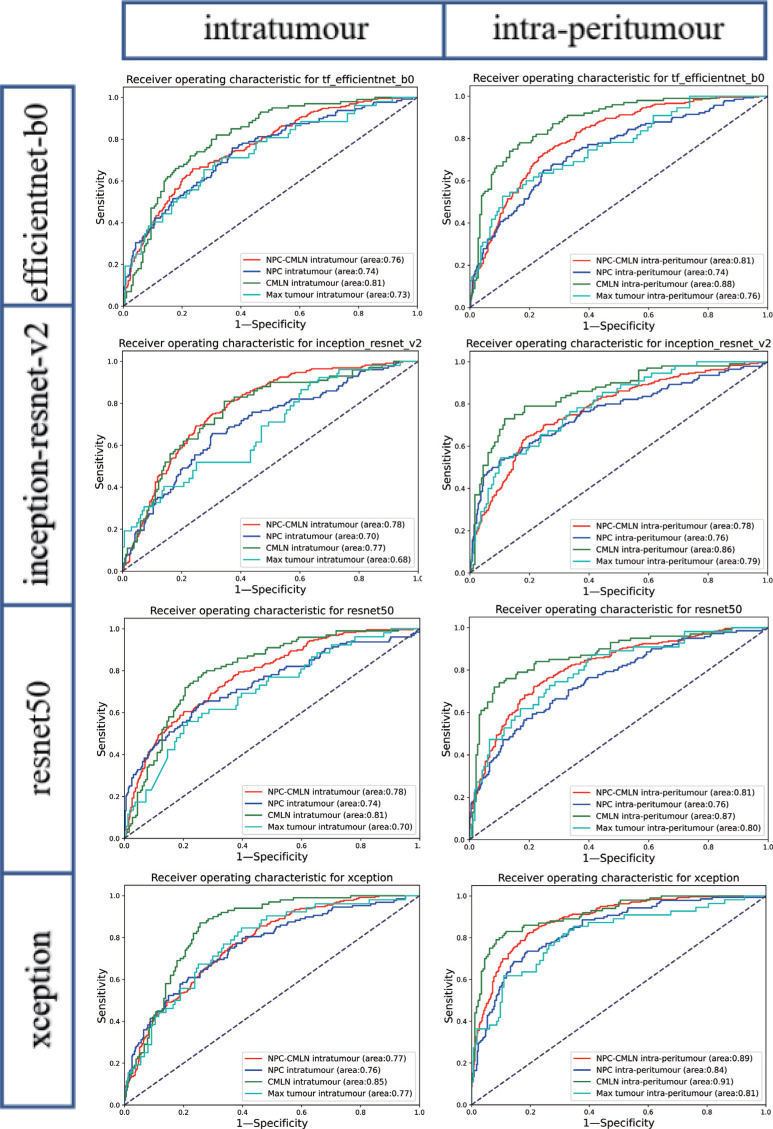
Receiver operating characteristic curves of DL Models.

Compared to that of the TNM classification (AUC = 0.67), after the training of the four models, the eight datasets (NPC-CMLN intratumour, NPC intratumour, CMLN intratumour, Max tumour intratumour, NPC-CMLN intra-peritumour, NPC intra-peritumour, CMLN intra-peritumour, and Max tumour intra-peritumour) exhibited an increased ability to predict the occurrence of DM, with the average AUCs being 0.77 (0.76-0.78), 0.74 (0.70-0.76), 0.81 (0.77-0.85), 0.72 (0.68-0.77), 0.82 (0.78-0.89), 0.78 (0.74-0.84), 0.88 (0.86-0.91), and 0.79 (0.76-0.81), respectively. The performance of TNM classification is similar to that in previous studies [27-29]. Among the four models, the four intra-peritumour datasets performed better than the four corresponding intratumour datasets; the CMLN dataset exhibited the best performance (Fig. **3**).

**Fig. (4):**
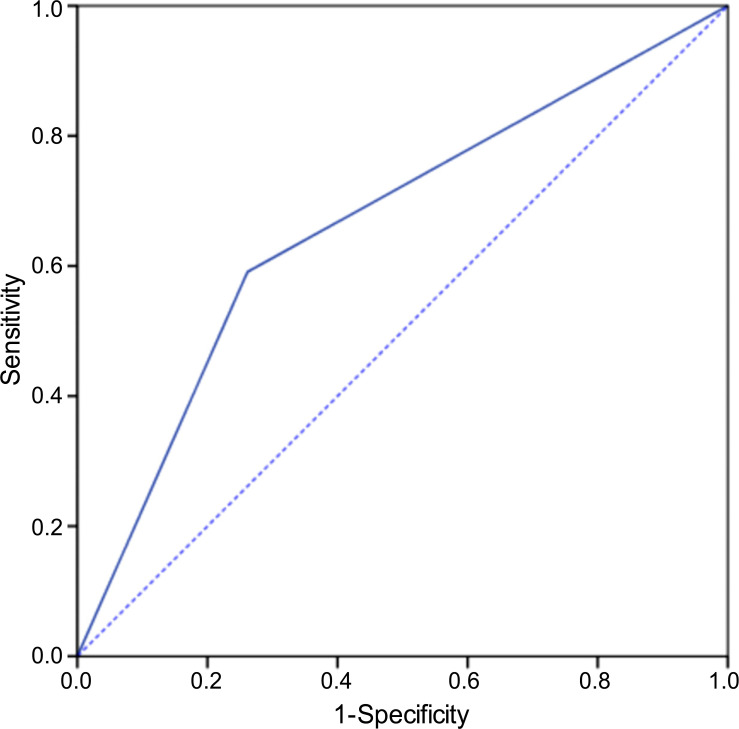
Receiver operating characteristic curve of the TNM staging system.

The average accuracy curve results of the NPC-CMLN intratumour dataset, NPC intratumour dataset, CMLN intratumour dataset, Max tumour intratumour dataset, NPC- CMLN intra-peritumour dataset, NPC intra-peritumour dataset, CMLN intra-peritumour dataset, and Max tumour intra-peritumour dataset were 74.17%, 69.40%, 74.91%, 68.45%, 75.82%, 75.22%, 80.39%, and 74.13%, respectively, which were higher than those of the TNM staging system (70.11%). The accuracy of the intra-peritumour datasets was higher than that of the four corresponding intratumour datasets, with the accuracy of the CMLN dataset being the highest (Fig. **4**). In addition to accuracy, sensitivity, specificity and F1-score of the models were calculated, which were similar to the ROC curve results Table **2**.

**Fig. (5):**
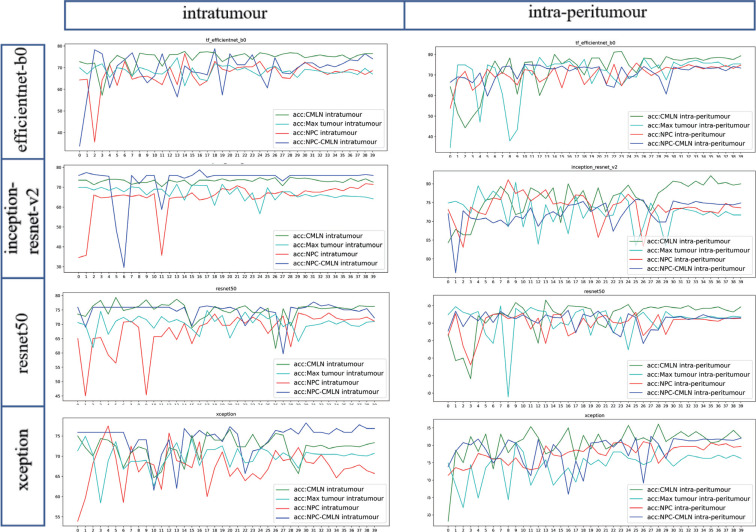
Accuracy curves of DL Models.

**Fig. (6):**
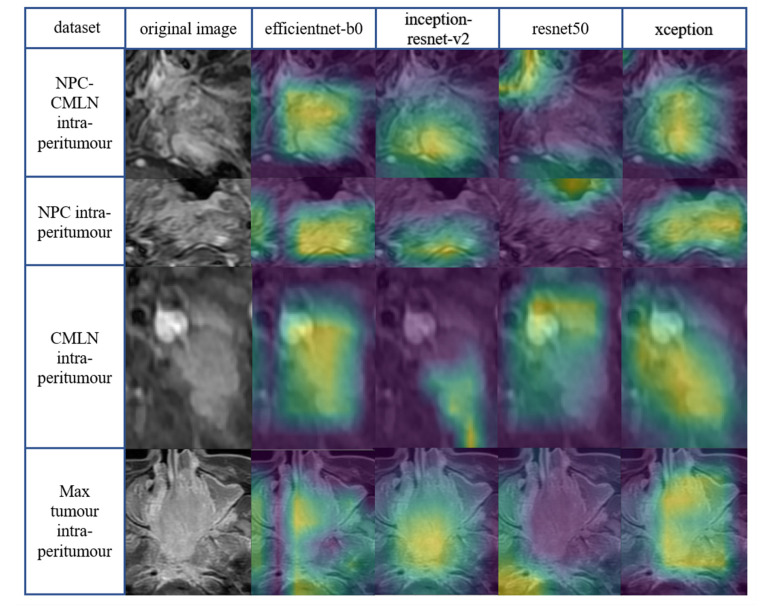
Grad-Cams of the DL Models in intra-peritumour datasets.

Class activation mapping is a powerful technique for computer vision classification tasks; it allows researchers to study the categorisation of images to better understand which parts of the image contribute the most to the model output. We used class activation mapping to generate Grad-Cams of yellow, green, and purple. The three colours of the Grad-Cams represented different degrees of predictive significance. The yellow region had the greatest correlation with classification, followed by the green and purple regions, which showed no predictive significance. The yellow and green areas for patients with DM represented characteristics that correlated with a higher metastatic risk. The presentation of the Grad-Cams aided a better understanding of how the DL network captured image features for prediction and resolved doubts regarding CNN's ability to learn in the appropriate direction. The yellow mainly distributed in the tumour region indicates the learning direction of the model is correct, while the concentration of the yellow area in the surrounding normal tissue area indicates the wrong learning direction. The more concentrated the yellow area is in the tumour region, the stronger the interpretability of the model. Efficientnet-b0, inception - resnet-v2, and xception models extracted high-risk features mainly concentrated in the tumour region, with efficientnet-b0 and xception being more concentrated. However, the high-risk features extracted by the resnet50 model deviated from the tumour region. Compared to the four intratumoural datasets (Fig. **5**), the Grad-Cams of the four intra-peritumoural datasets were more focused on tumour regions (Fig. **6**).

**Fig. (7):**
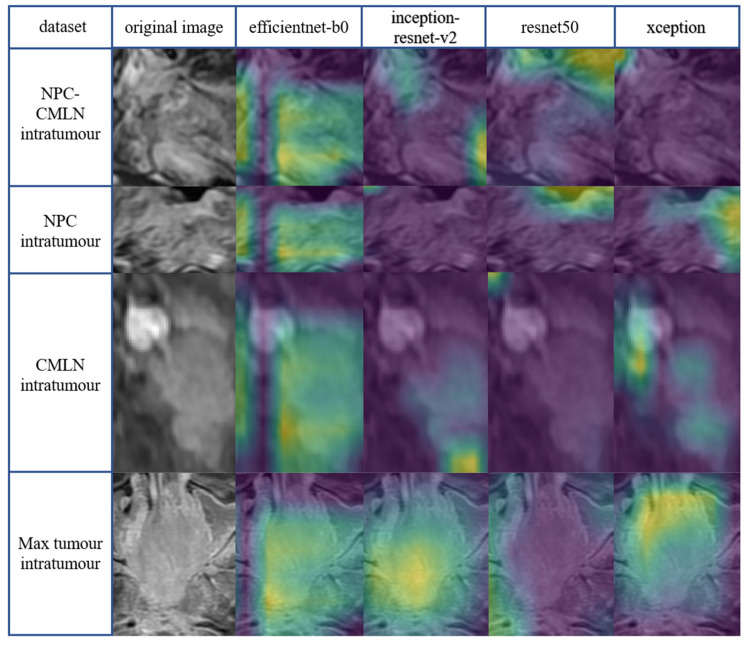
Grad-Cams of the DL Models in intratumour datasets.

**Fig. (8):**
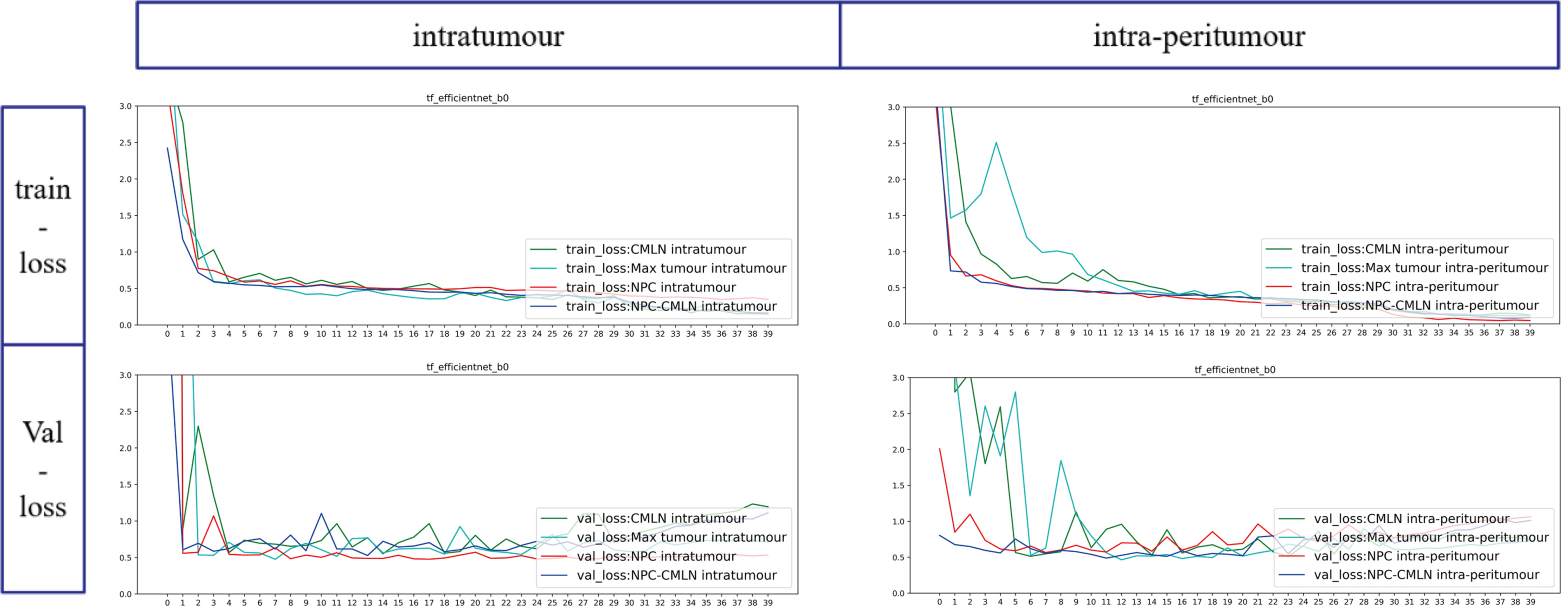
Loss function curves of the DL Models.

The loss function value in the model training process evaluates the degree of inconsistency between the predicted value and real value of the models and is a standard used to measure the quality of model prediction-the smaller the loss function, the better the model’s robustness. We used the efficientnet-b0 model as an example (Fig. **7**). With iteration of the epoch, there was a constant decrease in training and test losses, indicating that our model was constantly learning. In both the intratumoural and intra-peritumoural datasets, the CMLN and NPC-CMLN datasets had the smallest loss values (Fig. **8**).

## 4 DISCUSSION

The assessment of medical images is not limited to qualitative diagnosis but includes the acquisition and analysis of rich quantitative information providing data on disease severity, best treatment choice, and patient prognosis [30, 31]. Artificial intelligence plays an increasingly important role in this process because of its powerful feature extraction and screening abilities. The traditional evaluation of tumour images relied on qualitative characteristics, such as tumour densities, enhancement patterns, regularity of tumour margins, and relationship with surrounding tissues. However, the more “hidden” information regarding factors, such as patient prognosis, late DM, and response to specific drug therapies, could not be identified. As a subset of artificial intelligence, DL retrieves this “hidden” information because of its superior ability to automatically learn image features. This is a convenient way for researchers and clinicians to improve the accuracy of assessments and provide tailored medical services. With the gradual application of intensive radiotherapy in clinical practice, the risk of local recurrence in NPC patients has shown an increased level of control, with DM becoming the main cause of death in these patients [32, 33]. Although additional induction chemotherapy can, to a certain extent, reduce the risk of DM in some patients, it also significantly increases the incidence of adverse events [34, 35]. If the risk of DM can be predicted before radiotherapy and chemotherapy, intensive treatment can be selected for those at high risk of DM, ensuring the best treatment plan in the high-risk group while minimising the risk of serious adverse reactions, due to additional radiotherapy and chemotherapy, in the low-risk group.

Currently, treatment measures are formulated mainly based on the tumour’s TNM stage. However, in our study, the AUC and accuracy of the TNM stage in predicting the risk of DM in patients were only 0.67 and 70.11%, respectively. In a previous study on cervical cancer, the peritumoural region was found to play a role in predicting lymph node metastases [36]. Therefore, we also explored whether the information in the tumor tissue predicted DM. Compared to those of the NPC-CMLN intra-peritumoural, NPC intra-peritumoural, CMLN intra-peritumoural, and Max tumour intra-peritumoural datasets, the average AUCs of the NPC-CMLN intratumoural, NPC intratumoural, CMLN intratumoural, and Max tumour intratumoural datasets decreased from 0.82 to 0.77, 0.78 to 0.74, 0.88 to 0.81, and 0.79 to 0.72, respectively, suggesting that the peritumoural region plays a role in predicting DM in NPC patients. Our results showed that in both the intratumoural and intra-peritumoural datasets, the CMLN datasets had the highest AUCs, indicating that the CMLN had a higher predictive value for DM than the primary tumour of NPC. Moreover, comparing the CMLN datasets, the number of images in the NPC-CMLN datasets was significantly larger; however, the AUCs were lower than those of the CMLN, which further suggested that CMLNs are more significant in predicting DM than the primary tumour of the NPC. The AUCs of the Max tumour datasets were similar to those of the NPC datasets; however, the number of images comprising the Max tumour datasets was significantly lower than those of the NPC datasets because the Max tumour datasets were composed of only 3 images in each patient, and the number of images in the training cohort was < 1000, which was far lower than that of the NPC datasets. Accuracy may have been limited by the small number of images in the dataset. The dataset with slices of the largest invasion area may provide meaningful and accurate values with larger sample size.

Since different models have different data preferences, we chose four commonly used network models to train the datasets, thus avoiding some limitations of the models. We compared the average values of each dataset among the four models to obtain more stable and reliable results. By analysing the Grad-Cams generated by the four CNN models, we observed that the high-risk features captured by the efficientnet-b0and xception models were within the tumour region. The efficientnet-b0 and xception models’ Grad-Cams were more concentrated, indicating the correctness of the above models’ learning, while the resnet50 model extracted features that deviated from the tumour region. This was because each model had different data processing methods with different features being captured. Therefore, we trained multiple network models in the training model and selected the one most suitable for our data, according to its extracted features and accuracy. After comparing the above characteristics of the four models, we found that the xception model performed best. Compared to the four intratumoural datasets, the Grad-Cams of the four intra-peritumoural datasets were easier to interpret. This further indicated that the intra-peritumoural datasets better-predicted metastasis. Previous publications on DM of NPC were radiomics-based studies with very limited sample sizes [6, 22, 23]. Recently, a DL-based study attempted to predict DM of NPC. The authors included 186 NPC patients; however, their dataset comprised only image slices showing the maximum tumour extension. Thus, less than 200 images constituted the dataset; hence, the AUC value of their constructed model was only 0.66 [24]. Compared to previous studies, the advantages of our study are mainly reflected in the following three aspects: 1) The diversity of the data sets’ construction methods, *i.e.*, we compared the roles of the primary tumour to CMLN, intratumoural to peritumoural regions, and all slices of the tumour to three slices, showing the maximum tumour extension in predicting distant metastasis; 2) multiple network models were selected to balance the data preferences of individual models; 3) the sample size of images included in the present study was significantly larger than those in previous studies.

Although our DL model exhibited good predictive performance, our study had some limitations. First, our cohort size was relatively limited, but to ensure the quality of the dataset, we strictly excluded 1,135 patients from the initial list, leaving only 441 patients. Furthermore, given that EB virus DNA, lactic dehydrogenase, and haemoglobin levels are predictors of DM [37], using the DL to integrate these data and information from MR images to form a hybrid model is likely to obtain better predictive accuracy. Unfortunately, the technical restrictions of our medical record system prevented the retrieval of clinical laboratory data; thus, we were unable to implement this mixed DL model.

## CONCLUSION

Our study demonstrated that a CNN that analyses pre-treatment MR images could accurately predict DM in NPC patients. Our findings also indicated that both tumour regions and tissues surrounding the tumours could provide important information for predicting DM. Compared to the primary tumour, the CMLN showed better performance in predicting DM, suggesting that CMLN may be riskier than invasions of primary sites of the NPC.

## Figures and Tables

**Table 1:** Clinical characteristics of patients in the training and test cohorts.

**-**	**Training Cohort**		**-**	**P**	**-**	**Test Cohort**		**-**	***P***	***-***	***P***
	**DM**	**NO DM**	**-**	**-**	**-**	**DM**	**NO DM**	**-**	**-**	**-**	**-**
Patients	92	262	-	-	-	22	65	-	-	-	-
Age	50.93 ± 12.96	51.07 ± 11.02	-	0.92	-	50.45 ± 14.61	50.94 ± 11.29	-	0.87	-	0.88
Sex	-	-	-	-	-	-	-	-	-	-	-
Male	62	198	-	0.13	-	18	48	-	0.45	-	0.65
Female	30	64	-		-	4	17	-		-	
Staging^1^	-	-	-	-	-	-	-	-	-	-	-
I	0	7	-	0.00	-	0	5	-	0.00	-	0.55
II	1	47	-		-	0	7	-		-	
III	34	156	-		-	9	36	-		-	
IV	57	52	-		-	13	17	-		-	
T stage^1^	-	-	-	-	-	-	-	-	-	-	-
T1	7	32	-	0.00	-	2	7	-	0.08	-	0.46
T2	18	106	-		-	4	24	-		-	
T3	18	79	-		-	6	17	-		-	
T4	49	45	-		-	10	17	-		-	
N stage^1^	-	-	-	-	-	-	-	-	-	-	-
N0	3	40	-	0.00	-	1	10	-	0.00	-	0.76
N1	8	59	-		-	1	17	-		-	
N2	63	153	-		-	15	36	-		-	
N3	18	10	-		-	5	2	-		-	
Treatment	-	-	-	-	-	-	-	-	-	-	-
CCR^2^	37	143	-	0.02	-	9	39	-	0.12	-	0.47
IC+CCR^3^	55	119	-		-	13	26	-		-	

**Note:** ^1^Based on the 7th edition of the American Joint Committee on Cancer /International Union Against Cancer staging system.

^2^Concurrent chemoradiation.

^3^Including induction chemotherapy + radiotherapy, induction chemotherapy + concurrent chemoradiation CCR, concurrent chemoradiation; DM, distant metastasis; IC, induction chemotherapy.

**Table 2:** Area under the receiver operating characteristic curves and the accuracy of the DL Models and TNM staging system.

**Datasets**	**Models**		**AUC**				**Accuracy**			
			**Intratumour**		**Intra-Peritumour**		**Intratumour**		**Intra-Peritumour**	
NPC-CMLN	Efficientnet-b0	-	0.76	-	0.81	-	74.40	-	73.28	-
-	Inception-resnet-v2	-	0.78	-	0.78	-	76.04	-	74.78	-
-	Resnet50	-	0.78	-	0.81	-	72.27	-	72.95	-
-	Xception	-	0.77	-	0.89	-	76.68	-	82.25	-
-	-	Average	-	0.77	-	0.82	-	74.85	-	75.82
NPC	Efficientnet-b0	-	0.74	-	0.74	-	69.08	-	75.06	-
-	Inception-resnet-v2	-	0.70	-	0.76	-	71.20	-	73.62	-
-	Resnet50	-	0.74	-	0.76	-	71.20	-	72.95	-
-	Xception	-	0.76	-	0.84	-	66.11	-	79.25	-
-	-	Average		0.74	-	0.78	-	69.40	-	75.22
CMLN	Efficientnet-b0	-	0.81	-	0.88	-	76.81	-	79.58	-
-	Inception-resnet-v2	-	0.77	-	0.86	-	72.96	-	80.24	-
-	Resnet50	-	0.81	-	0.87	-	76.52	-	79.50	-
-	Xception	-	0.85	-	0.91	-	73.35	-	82.25	-
-	-	Average	-	0.81	-	0.88	-	74.89	-	80.39
Max tumour	Efficientnet-b0	-	0.73	-	0.76	-	67.42	-	75.38	-
-	Inception-resnet-v2	-	0.68	-	0.79	-	64.63	-	71.65	-
-	Resnet50	-	0.70	-	0.80	-	71.20	-	72.97	-
-	Xception	-	0.77	-	0.81	-	70.56	-	76.50	-
-	-	Average	-	0.72	-	0.79	-	68.45	-	74.13
TNM	-	-	0.67				70.11			

## Data Availability

The data supporting the findings of this study are available within the article.

## LIST OF ABBREVIATIONS

NPC: Nasopharyngeal Carcinoma
CNN: Convolutional Neural Network
CMLN: Cervical Metastatic Lymph Node
AUC: The area under the receiver-operating characteristic curve
DM: Distant Metastasis
DL: Deep Learning
ROI: Region of Interest
